# The Impact of Modifiable Environmental, Nutritional, and Lifestyle Factors on Puberty, Reproductive Function, and Fertility in Children and Adolescents

**DOI:** 10.3390/children10050781

**Published:** 2023-04-26

**Authors:** Valeria Calcaterra, Gianvincenzo Zuccotti

**Affiliations:** 1 Department of Internal Medicine and Therapeutics, University of Pavia, 27100 Pavia, Italy; 2 Pediatric Department, Buzzi Children’s Hospital, 20154 Milano, Italy; gianvincenzo.zuccotti@unimi.it; 3 Department of Biomedical and Clinical Science, University of Milano, 20157 Milano, Italy

The survival of a species depends on its ability to reproduce [1]. It has been estimated that up to 15% [2] of people worldwide may be infertile and various modifiable environmental, nutritional, and lifestyle factors that can affect fertility, particularly during the critical period from intrauterine development to adolescence [3].

Puberty is the stage when fertility is achieved and maintained throughout all subsequent stages of life. This process is initiated by the pulsatile secretion of the gonadotropin-releasing hormone (GnRH) from the hypothalamus [4,5]. GnRH secretion is fully active in the neonatal period, quiescent throughout most of childhood, and is reactivated at the time of puberty to induce sexual maturation and subsequent fertility [4,5]. 

Factors such as genetic predispositions, under or overnutrition, and exposure to environmental endocrine disruptors can influence the timing of puberty and affect the integrity of the hypothalamic–pituitary–gonadal axis, which is essential for preserving fertility at all stages of life [4,5]. Patients with disorders related to pubertal development are prone to developing gonadal dysfunction of various kinds, such as polycystic ovary syndrome at a young age, influencing the reproductive function and fertility [4,5].

Adolescence and young adulthood are critical stages when lifestyle factors such as weight, smoking, alcohol, caffeine consumption, diet, exercise, psychological stress, and environmental and occupational exposures can impact fertility in both sexes [6,7,8]. 

For example, obesity and being underweight are associated with gonad dysfunction and infertility, while certain vitamins (including vitamins B, C, and E) and food groups (including high-fat diets) have an important impact on reproductive health by influencing semen quality and ovulation [9,10,11,12,13]. Smoking, alcohol, and caffeine consumption can interfere with fertility and affect gonad function and reserve and influence endocrine function (FSH, LH, and testosterone in males) [1,14,15,16,17,18,19]. Although the relationship between stress and infertility has not yet been fully elucidated, and it is uncertain which is the cause and which is the effect, physical stress has also been implicated in influencing reproductive function and environmental and occupational exposures, such as air pollution, heavy metals, pesticides, endocrine disruptors, and other chemicals, and radiation can negatively impact fertility by affecting sensitive cells in the body, including germ cells [20,21].

Additionally, excessive exercise can affect body energy balance and hypothalamic function, negatively impacting the reproductive system [4,5]. 

Finally, unhealthy lifestyle and environmental exposures can have transgenerational effects on endocrine disruptors in reproductive tissues, not only through oocytes, but also through sperm [22], demonstrating the importance of exploring this topic further.

Understanding the impacts of environmental, nutritional, and lifestyle factors in regulating the reproductive system is essential for maintaining gonadal function, preserving fertility and protecting overall wellbeing. 

Preventative strategies that modify lifestyle factors can be useful in enhancing reproductive health and maximizing fertility outcomes, starting from childhood.
